# A comparison of experience sampled hay fever symptom severity across rural and urban areas of the UK

**DOI:** 10.1038/s41598-023-30027-x

**Published:** 2023-02-21

**Authors:** Ann Gledson, Douglas Lowe, Manuele Reani, David Topping, Ian Hall, Sheena Cruickshank, Adrian Harwood, Joshua Woodcock, Caroline Jay

**Affiliations:** 1 Research IT, University of Manchester, Manchester, UK; 2 School of Management and Economics, The Chinese University of Hong Kong, Shenzhen, China; 3 Department of Earth and Environmental Sciences, University of Manchester, Manchester, UK; 4 Department of Computer Science, University of Manchester, Manchester, UK; 5 Department of Mathematics, University of Manchester, Manchester, UK; 6 Division of Infection, Immunity and Respiratory Medicine, University of Manchester, Manchester, UK

**Keywords:** Environmental chemistry, Quality of life, Immunology, Signs and symptoms

## Abstract

Hay fever affects people differently and can change over a lifetime, but data is lacking on how environmental factors may influence this. This study is the first to combine atmospheric sensor data with real-time, geo-positioned hay fever symptom reports to examine the relationship between symptom severity and air quality, weather and land use. We study 36145 symptom reports submitted over 5 years by over 700 UK residents using a mobile application. Scores were recorded for *nose*, *eyes* and *breathing*. Symptom reports are labelled as urban or rural using *land-use* data from the UK’s Office for National Statistics. Reports are compared with AURN network pollution measurements and pollen and meteorological data taken from the UK Met Office. Our analysis suggests urban areas record significantly higher symptom severity for all years except 2017. Rural areas do not record significantly higher symptom severity in any year. Additionally, symptom severity correlates with more air quality markers in urban areas than rural areas, indicating that differences in allergy symptoms may be due to variations in the levels of pollutants, pollen counts and seasonality across *land-use* types. The results suggest that a relationship exists between urban surroundings and hay fever symptoms.

## Introduction

The worldwide prevalence of allergic respiratory disease has risen considerably in recent years^1^. Whilst air pollution is considered to worsen symptoms for the individual^2–7^, increase pollen concentrations, and lengthen pollen seasons^8^, the mechanisms of these combined effects on symptom severity are still not fully understood.

To investigate the relationship between air quality and hay fever symptoms, this paper reports the first study to compare the severity and duration of real-time symptom reports across rural and urban areas using experience sampled, geo-positioned cross-sectional data^9^. The use of mobile application data to collect users’ symptom reports for comparison with environment data has increased over the last few years. Peeters et al. used two years of geo-positioned mobile app data to compare chronic rhinosinusitis symptoms with air pollution data in Belgium. They found that, during the spring/summer months, a relationship existed between symptoms and exposure to $\hbox {O}_3$ and $\hbox {PM}_{2.5}$^4^. Cabrera et al., using 2 years of seasonal allergic rhinitis symptom data recorded in Madrid, found that temperature and pollution (most significantly $\hbox {O}_3$), out of all the environment indicators investigated, had the highest association with participant symptoms^10^. Kim et al. discovered an association between allergic rhinitis and $\hbox {SO}_2$ in a cohort of elementary school children in an industrial region of Korea^5^.

We hypothesise that people experience more severe symptoms in urban than in rural areas, due to an increase in the immune system burden. Urban and rural regions are reported to vary in pollen counts and types^11^, pollution levels^12–15^, rates of allergic reactions^16–22^ and daily mortality rates^23^:

### Hypothesis 1

(H1) Seasonal allergy symptoms are more severe for those in urban areas than in rural areas.

We also investigate whether higher pollution levels are related to more severe seasonal allergy symptoms:

### Hypothesis 2

(H2) Higher levels of pollution lead to worse seasonal allergy symptoms.

The Britain Breathing (BB) mobile application^24^ supports the collection of experience sampled, cross-sectional hay fever symptom data from the general population. Developed for Android and iOS, and made available through Google Play and the Apple App Store, we used it to recruit a large sample of *citizen scientists* who live in various locations throughout the UK, and are interested in contributing towards research into hay fever symptoms^9,25^. Previous studies of allergy symptoms have compared environmental data with medical data including asthma hospitalisation counts^1^, epidemiological studies of asthma^26^ and allergy questionnaires^27,28^. In this study, data is collected from those experiencing a range of hay fever symptoms from mild to severe, thus including people who experience common allergy symptoms but do not present to medical services. This provides a broader picture of chronic health issues experienced by hay fever sufferers, as opposed to only observing those with more acute and/or problematic reactions. Within the last decade, mobile applications have increasingly been used to collect experience sampled data from the general population for public health studies, as they are popular with users, allowing easy recruitment^4,25,29^. They are often convenient to use and maintain, lowering the cost of user support, regardless of the number of participants, and potentially improving participant engagement. In addition, the resulting data is available for analysis as soon as it is entered by the user.

**Figure 1:**
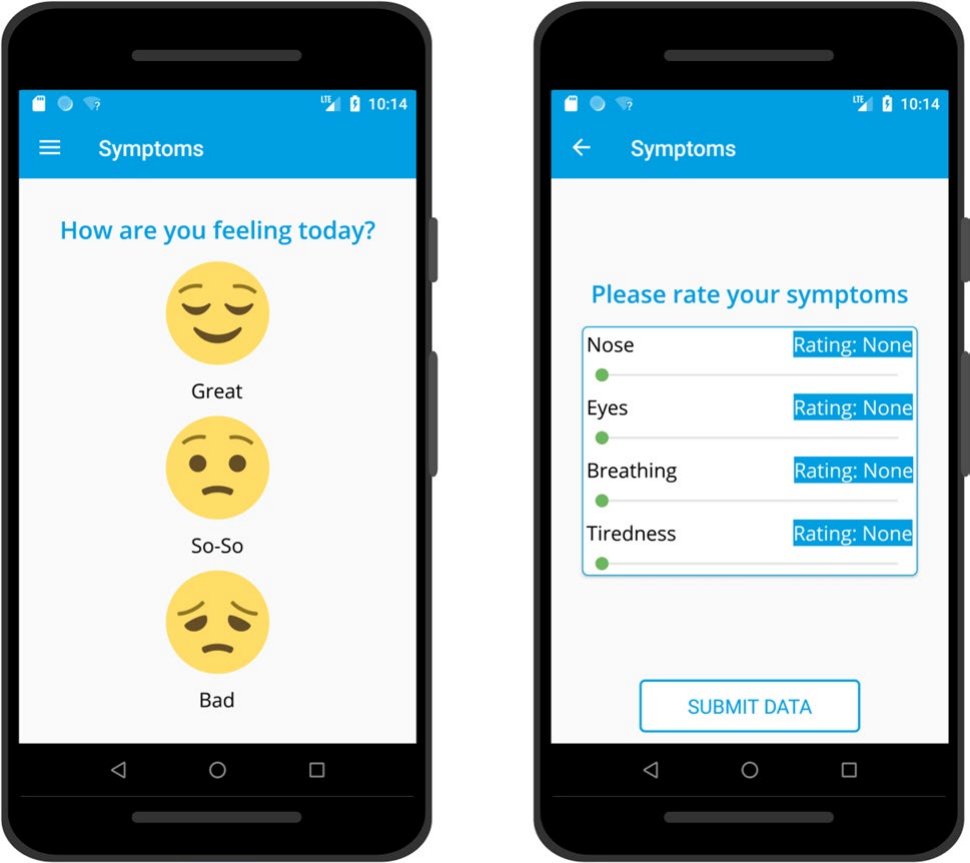
The BritainBreathing web application example screenshots.

Figure 1 displays screenshots from the data collection pages of the BB application, which gathers information about a user’s current condition. It asks whether they have taken any medication for their symptoms that day, and uses sliders to capture the severity of four symptoms: *nose*, *eyes*, *breathing* and *tiredness*. As *tiredness* was only added in a later version of the app, we focus in this analysis on *nose*, *eyes* and *breathing*. The sliding scale allows submission of the scores: 0 (no symptoms), 1 (mild symptoms), 2 (moderate symptoms) or 3 (severe symptoms). The time, date and location of each report is logged, providing symptom data at a level of temporal and spatial precision not captured by the medical record, questionnaire or prescription data commonly used for studying allergies. Location data is provided by the mobile device’s GPS sensor when the user permits it. Accuracy is set to 100–500 m to provide sufficient precision for the purposes of the study while complying with our data protection and ethical obligations.

A study conducted for 6 months over March–October 2016 showed that the experience sampling method used in the BB mobile application is *a reliable approach for collecting allergy symptom data in the general population*^9^. In this study we use data collected via the BB mobile application^9^ from 2016 to 2020 to compare allergy symptoms reported in urban and rural locations over this 5 year period.

## Methods

### Overview

We primarily investigate whether any significant differences in symptom severity exist between BB application users in urban and rural locations. The allergy symptoms measured are *nose*, *eyes*, *breathing* and *max score* (a calculation of the maximum of the former 3 scores). Each score is an integer in the range 0 to 3, with 3 being the most severe. The user is also asked if they have taken any allergy medications that day (possible answers are *yes* or *no*).

Secondly, we test for urban/rural differences in the correlations between each of these symptoms and a variety of air quality and meteorology measurements: $\hbox {PM}_{2.5}$, $\hbox {PM}_{10}$, $\hbox {NO}_2$, $\hbox {NO}_X$ (as $\hbox {NO}_2$), $\hbox {SO}_2$, $\hbox {O}_3$, relative humidity, temperature, air pressure and 12 pollens.

### Data collection

#### Britain breathing data

The Britain Breathing study was approved by the University of Manchester Ethics Committee, number CS 250 and carried out in accordance with all UK guidance and regulations. Informed consent was obtained from all participants at the start of the study.

The Britain Breathing mobile application was first released as an Android app on the Google Play store on March 18, 2016^9^ and this version continued until October 30 2016, and then was used again in March to October in 2017 and 2018. A second version was released in 2019 and also included an Apple version, made available on the Apple Store. The BB project and app were advertised *via social media, blogs, websites, public engagement activities, appearances in science festivals and on public television*^9^.

At the time that the user installs the application, they are asked for basic demographics such as gender, age and *do you have hay fever?* Participants are asked to report their symptoms each day for each of the three symptoms, using a simple sliding scale widget, designed to allow easy selection of one of the available 4 (0 to 3) scores and whether they have taken allergy medication that day (see Fig. 1). Each time the user inputs their scores, they are sent to a central server and recorded along with the date, time and the phone’s geographical location at the time of submission. In order to make the data non-sensitive, user identifiers were not included in the 2016 dataset, but they were included during subsequent years.

#### Britain breathing participants

At the end of October 2016, the app had been *downloaded 1530 times, 425 people had the app installed on their phones* and 20278 reports had been submitted^9^. In the years 2017 to 2020, 924 users had downloaded the application and 17,526 daily reports were submitted.

To rule out any bias relating to user demographics across rural and urban locations, participant data was analysed for gender, age and allergy medication usage. The female to male ratio in urban locations is 0.944 and in rural locations 0.927. The mean user age for all urban reports is 51.1 and for rural reports 57.3. Finally, the mean value of those who had taken medication for allergies on the same day as the report submission was 0.57 for urban locations and 0.54 for rural.

#### Land-use data

*Land-use* data was obtained from the UK’s Office for National Statistics (ONS)^30^ and used to divide the user reports into *rural* and *urban*, using the geographical co-ordinates recorded at the time of report submission. The ONS *2011 Census Rural–Urban Classification*^30^ categories are described in the Supplementary Appendix. Categories A1, B1, C1, and C2 for England and Wales, and 1, 2, and 3 for Scotland, are classified as *urban*. All remaining categories are classified as *rural*.

#### Environment data

Total daily pollen grain counts were available from UK monitoring sites for the 12 pollen types: hazel (*Corylus* spp., 13 sites), alder (*Alnus* spp., 13 sites), willow (*Salix* spp., 13 sites), birch (*Betula* spp., 13 sites), ash (*Fraxinus* spp., 13 sites), elm (*Ulmus* spp., 13 sites), oak (*Quercus* spp., 13 sites), plane (*Platanus* spp., 13 sites), grass (Poaceae, 15 sites), nettle family (Urticaceae, 13 sites), mugwort (*Artemisia* spp., 13 sites), and ragweed (*Ambrosia* spp., 13 sites). The data collection period for the pollen count monitoring stations is early March to early September in the years 2016 to 2020, and the data are obtained from the UK Met Office (MIDAS dataset) via the MEDMI server. Hourly measurements of $\hbox {PM}_{2.5}$ (81 sites), $\hbox {PM}_{10}$ (81 sites), $\hbox {NO}_2$ (161 sites), $\hbox {NO}_X$ (as $\hbox {NO}_2$, 161 sites), $\hbox {SO}_2$ (28 sites), and $\hbox {O}_3$ (75 sites) were downloaded from the Automatic Urban and Rural Network (AURN) network. Hourly measurements of relative humidity (323 sites), temperature (323 sites), and air pressure (154 sites), are also obtained from the UK Met Office (MIDAS dataset) via the MEDMI server.

### Pre-processing

#### Britain breathing data

BB data was collected as a CSV file with each row representing a user report submission and each field containing report information such as time, location and symptom scores. Several pre-processing steps were performed on the symptom data before all subsequent analysis: *For 2016 data, user identifiers were improvised using year-of-birth, gender and postcode location. See the Methodological Limitations Section for the potential affects of this.* Reports were filtered to include only those submitted within the months March to September inclusive. Only the latest report per day per user and only reports from users who submitted on at least 10 days, were used. This left 11,576 reports by 344 users for 2016; and 11,662 reports by 417 users for the years 2017 to 2020.

Each report (row) was assigned a postcode, firstly by inputting the geographical co-ordinates into a postcode finder API^31^. If no postcode was found (approximately 10% of reports), the location co-ordinates were mapped to the closest location found in a further online location-to-postcode mapping tool^32^. Reports were then labelled as urban or rural, using the ONS postcode classifications and the *max_symptom* score was calculated, which is the maximum of all 3 symptoms (*nose*, *eyes* and *breathing*).

#### Environment data

The environment measurements consisted of daily means and maximums for each of the pollutants and meteorological variables (calculated from hourly data) and daily counts for the pollen variables. The pollutant and meteorological variables were cleaned, and missing hourly values were (where appropriate) imputed before the daily means and maximums were calculated. We have made all of the pre-processed pollen, pollutant and meteorological sensor data described above publicly available at^33,34^. The cleaning and imputation methods used are described in^35^ and the pre-processing tools are available at^36^.

#### Regional estimation of environment data using concentric regions

To link each BB symptom report to environment variables, a regional estimation method is used. We started with the requirement that we needed to preserve the anonymity of the study participants while, at the same time, linking their reported symptoms with atmospheric measurements. Postcode regions were selected for the estimations as they provide similar population sizes, are large enough to provide anonymity and they have clearly defined geographic areas which can be estimated using nearby sensor data. If one or more sensors exist in the same region as the BB report, the mean is used. If not, a *concentric regions* method^35^ is used to find environment measurements from the closest possible regions. This searches for sensors in those postcode regions directly adjacent to the reporting region and if none are found, searches the next ring, until sensors are found, from which the mean is taken. We have made all of the pollen, pollutant and meteorological sensor regional estimations publicly available at^37,38^. The regional estimations methods used are described in^35^ and the tools used to pre-process are available at^39^.

### Methodological limitations

The methods used in this study do have some limitations, which should be noted here. Firstly, all participants included in the study are self-selected and therefore are unlikely to represent a random sample of the population. For example it is hypothesised that those who downloaded the BB mobile application and regularly recorded symptoms are more likely to be hay fever sufferers.

Another limitation is the inability to measure the effect of taking medication (using the ’Taken Medication?’ response on the BB mobile app) on the outcomes of this study. This is because there is no record of whether participants reported symptoms before or after taking any antihistamines. For this reason, we do not stratify results by the responses to this question, but only use it as a potential extra indicator of symptom severity.

Participants were asked to report once-per-day, but as no limit was set by the mobile application, we selected the latest report for each day, for each participant. User identifiers are present in the 2017–2020 data, but for the 2016 data user identifiers had to be improvised using year-of-birth, gender and postcode location. This will result in multiple reports being included for users that reported from different postcodes on the same day. To a lesser extent, it could also result in multiple users who reported from the same postcode, with the same gender and year-of-birth being treated as a single user. Although we cannot quantify the scale of either of these anomalies, it is not expected that they would significantly effect the comparisons between urban and rural symptom severity reports.

## Results

Within this section we split the analysis of our dataset into three stages. First, we compare symptom severity and duration in urban and rural areas, finding that symptom durations are longer, and severity higher, in urban areas for four of the five years studied. Secondly, we explore symptom and environmental data correlations for urban and rural areas for the whole of the UK, finding that correlations between symptoms and pollutants are strong, with relationships more likely to be found in urban than rural areas. Finally, we explore relationships between symptom and environmental data at the regional level, using postcode areas for matching data, and the *concentric regions* method for filling gaps in the environmental dataset^35^ (see “Methods”, “Pre-processing” section). These regional correlations are found to be weak, which may be due to the complexity of interactions between pollutants and bio-aerosols^40^ and the variability of human biological response to those interactions^20^, and/or difficulties accurately sampling environmental data at this fine granularity.

The BB *n* values (user and report counts) for each year, used for all results are as follows (note that 2016 user counts are calculated using derived user IDs, as described in the “Methods”: “Pre-processing” section): urban-2016 285 users, 9543 reports; urban-2017 133 users, 4028 reports; urban-2018 84 users, 3094 reports; urban-2019 30 users, 697 reports; urban-2020 15 users, 778 reports; rural-2016 59 users, 2033 reports; rural-2017 78 users, 1073 reports; rural-2018 50 users, 1294 reports; rural-2019 19 users 424 reports; rural-2020 8 users, 274 reports.

The environmental data used are pollutant measurements sourced from the Automatic Urban and Rural Network (AURN), and meteorological and pollen measurements from the UK Met Office (MIDAS dataset)^33,34^. More details of these measurements, and how they are preprocessed, are included in the Methods section. For all analyses comparing BB reports with environmental data, only data from the months March to September (inclusive) are used, as this is when pollen data is collected and pollen allergies are strongest. In all of our analyses, only the latest report per day, per user is included. To avoid including highly disengaged users, only reports from users who have submitted on $\ge$ 10 days are used. We perform a between-subjects analysis at the level of symptom reports, as the sporadic reporting that is common in longitudinal citizen science studies and the fact that people tend to report from either urban or rural areas, rather than across both, means that an inferential within-subjects analysis at the level of the participant would be unreliable.

We explore the differences between urban and rural symptom reports as these two location types act as abstract intermediaries for representing / grouping the complex interactions between the different environmental factors and the reported hay fever symptoms. We label the BB symptom scores as urban or rural using ONS land-use classifications (see the Methods section for the criteria used).

### UK-wide urban vs rural symptom severity

To investigate the first hypothesis (H1), we compared mean daily symptom severity between land-use types. Reports were classified as urban or rural and we calculated the mean scores for each day, for each location type and symptom combination. These mean scores are then aggregated by year (2016 to 2020). Table 1 shows comparisons between urban and rural mean scores for each symptom (or whether medication was taken), for all months of the year. (See Supplementary Table S1b for comparisons using only BB reports from March-September incl., in which the differences are as pronounced, if not more so.) The first row displays the averages across all years, and the remaining rows display each year. Nested rows show data for each symptom. The *diff mean* column is the *urban mean* score minus the *rural mean* score, so positive values indicate a higher urban mean score. The table shows that, when averaging across all years, symptoms reported from urban locations have a considerably higher severity. For an expanded version of this table which includes urban and rural standard deviations, see Supplementary Table S1a. We used *Cohen’s d*^41^ to measure the effect size between the two means. Effect sizes can be categorised as: 0.01 = very small; 0.2 = small; 0.5 = medium; 0.8 = large; 1.2 = very large; 2.0 = huge^42^. Table 1 also displays the non-parametric *Kolmogorov–Smirnov* test result (U1), used to compare the distance between the urban and rural daily mean distributions. Higher scores represent a greater difference between the two distribution functions, with a possible range of 0 to 1. Each individual year shows considerably higher severity in urban areas in at least one symptom, except in 2017, where no substantial differences exist. Rural areas record no considerably higher symptoms than urban areas (no increases greater than 0.061), in any year. The overall results indicate generally higher symptom severity scores in urban regions, supporting H1.

**Table 1:** Urban vs rural symptom severity (all months).

	BB measure	Urban mean	Rural mean	Diff mean	Cohen’s d	U1	p (2-tailed)
All years	Nose	0.738	0.376	0.362	0.887	0.406	<.001
	Eyes	0.614	0.372	0.241	0.57	0.352	<.001
	Breathing	0.63	0.331	0.299	0.7	0.293	<.001
	Taken_meds	0.63	0.397	0.233	0.818	0.310	<.001
	Max_score	1.037	0.614	0.423	0.843	0.35	<.001
2016	Nose	0.566	0.315	0.251	1.2	0.538	<.001
	Eyes	0.429	0.296	0.133	0.692	0.396	<.001
	Breathing	0.336	0.389	-0.052	-0.338	0.271	<.001
	Taken_meds	0.496	0.51	-0.014	-0.102	0.196	<.001
	Max_score	0.786	0.649	0.136	0.592	0.342	<.001
2017	Nose	0.626	0.6	0.027	0.09	0.136	.0223
	Eyes	0.484	0.541	-0.057	-0.202	0.185	<.001
	Breathing	0.398	0.459	-0.061	-0.253	0.174	.0013
	Taken_meds	0.574	0.607	-0.032	-0.163	0.149	.009
	Max_score	0.911	0.918	-0.007	-0.021	0.142	.0147
2018	Nose	0.705	0.592	0.113	0.352	0.169	<.001
	Eyes	0.598	0.604	-0.006	-0.015	0.093	.0795ns
	Breathing	0.57	0.545	0.025	0.077	0.112	.019
	Taken_meds	0.703	0.593	0.11	0.55	0.287	<.001
	Max_score	1.044	0.931	0.113	0.271	0.177	<.001
2019	Nose	0.593	0.28	0.314	0.713	0.423	<.001
	Eyes	0.5	0.349	0.151	0.321	0.349	<.001
	Breathing	0.668	0.195	0.473	0.959	0.429	<.001
	Taken_meds	0.654	0.282	0.372	1.142	0.435	<.001
	Max_score	0.972	0.443	0.529	0.897	0.428	<.001
2020	Nose	1.057	0.12	0.937	2.123	0.821	<.001
	Eyes	0.957	0.1	0.857	1.975	0.815	<.001
	Breathing	0.963	0.133	0.83	1.769	0.772	<.001
	Taken_meds	0.625	0.095	0.53	2.036	0.754	<.001
	Max_score	1.287	0.215	1.072	2.205	0.784	<.001

Average daily scores for each are presented, with the difference between the two (diff), the Kolmogorov–Smirnov test statistic (U1), and significance (*p*). *p* values > 0.05 are marked as *ns*.

**Figure 2:**
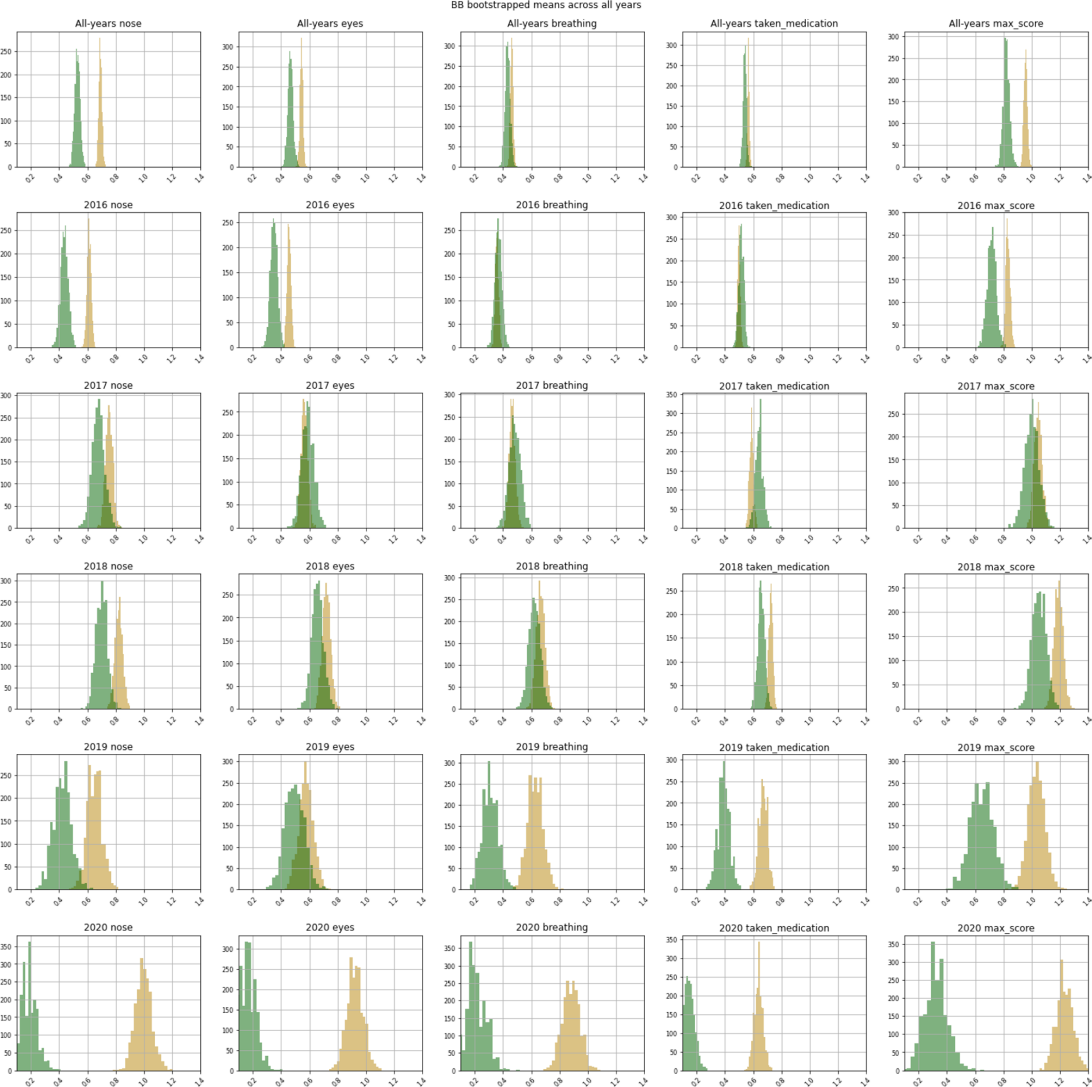
Urban (gold) vs rural (green) bootstrap re-sampling of average symptom severity. Each column presents a single symptom (left to right: nose, eyes, breathing, taken medication and max score). Each row contains a different year (top to bottom: all years, 2016, 2017, 2018, 2019 and 2020). Y-axes represent counts, x-axes represent mean scores. Each sample size is 20% of original data, and 2000 different samples were used for this analysis.

As the above comparisons were not performed as part of a controlled study, it is necessary to check for any indication that the positive results are biased, for example by very high numbers of reports from individuals in one land use type. To achieve this, we used a re-sampling bootstrap test to repeat these comparisons multiple times on smaller samples: 2000 random samples (each containing 20% of the total number of BB reports) were taken and the mean of each sample calculated and plotted in a histogram. Figure 2 displays each of the resulting histograms. Each year is displayed in a row (the top row represents all years together) and the columns show *nose*, *eyes*, *breathing*, *medication taken* and *max_score*. The gold histograms represent the distributions of urban means and green represent rural. The results illustrate the strength and consistency of the differences between urban and rural means for all symptoms in 2020. Other years show more mixed urban/rural differences, although the differences are still clear with the exceptions of breathing in 2016, nose and max score in 2018 and eyes in 2019. For data across all years (top row), these differences are clear for nose, eyes and max score. As we see the same effect when using this re-sampling method, we can be reasonably confident that it isn’t biased by a few individuals in one group.

### UK-wide urban vs rural symptom duration

To further test the validity of H1, we examined the duration of reports with symptoms scores greater than 0 (meaning that at least some symptoms were experienced) for each user, also allowing for single non-reporting days. Results indicate that durations are slightly longer in urban areas than in rural, supporting H1. Table 2 shows the average duration in days for which users report higher symptoms from March to September inclusive, and compares the differences in duration between urban and rural users. The *rural* and *urban* columns show the average number of unbroken, *chains* of higher scoring days per participant. Note that 2016 data has derived user IDs, as described in the “Methods”: “Pre-processing and methodological limitations” sections; as a result of this, chains of symptom reports are more easily broken in this cohort, as reports by the same user can appear to come from different users where they report from different postcode locations. It is not expected that this will affect urban and rural comparisons. The *min score* column shows the minimum score that must be recorded, for the chain to be unbroken. The *allowed gap* column shows the number of days that a user is allowed to miss (not submit *any* report), before the chain is broken. The results indicate that most urban symptom duration means are higher than rural, for chains of days with a minimum score of 1 or 2. No difference is recorded for chains of days with scores of only 3, as both medians are of 1 day chains only, for each symptom.

**Table 2:** Urban vs rural symptom duration: medians of the user average (mean) duration of symptoms in days (March–Sept incl.).

Min score	Allowed gap (days)	Symptom	Rural	Urban	Diff (urban − rural)
1	0	Breathing	1.188	1.286	0.098
		Eyes	1.25	1.333	0.083
		Max_score	1.4	1.5	0.1
		Nose	1.3	1.414	0.114
		Taken_medication	1.517	1.526	0.009
	1	Breathing	1.427	1.533	0.106
		Eyes	1.333	1.625	0.292
		Max_score	1.598	2	0.402
		Nose	1.5	1.75	0.25
		Taken_medication	1.858	2.125	0.267
2	0	Max_score	1	1.25	0.25
	1	Breathing	1	1.25	0.25
		Eyes	1.167	1.2	0.0339
		Max_score	1.286	1.4	0.114
		Nose	1	1.286	0.286
3	0	Max_score	1	1	0
	1	Max_score	1	1	0

Except for tests using maximum symptom score (*max_score*), all rows where the difference is zero are removed.

### UK-wide correlations for urban and rural locations

To explore the validity of our second hypothesis (H2) that higher levels of pollution lead to worse hay fever symptoms, the BB user reports are again divided into urban and rural locations and compared with environment measures using monthly averages. We use all UK sensors and take monthly averages, to discover if correlations exist at a general, coarse-granularity. All pollen and weather sensor data are used as they are not classified into location types, but for the pollutant dataset, we include only *urban background* and *rural background* sensor measurements. We do not use any sensor data from roadside or industrial locations as we are most interested in tracking large-scale regional patterns in pollution, and these could be masked by the local pollution events measured at these sites. We begin by correlating urban and rural symptoms with UK-wide averages for all environmental measures without dividing the pollution sensors into urban and rural locations (Table 3). To check if urban and rural reports are more correlated with their respective sensor types, we also look at correlations between monthly symptoms and pollution measurements, grouped by *urban background* and *rural background* sensor type (Table 4). (Note that this is not possible for non-pollutant variables, as some sensors are not classified by location type).

**Table 3:** Correlations of BB symptom scores with average monthly environment variables (UK-wide, March–Sept incl.).

Environment measure (UK)	BB symptom (urban)	Correlation	p (2-tailed)
$\hbox {SO}_2$ mean	Eyes	−0.7163	$\le$.001
$\hbox {NO}_x$ mean	Eyes	−0.6446	$\le$.001
$\hbox {SO}_2$ mean	Nose	−0.6248	$\le$.001
$\hbox {NO}_2$ mean	Eyes	−0.6009	$\le$.001
$\hbox {NO}_x$ max	Eyes	−0.6005	$\le$.001
$\hbox {NO}_x$ mean	Breathing	−0.596	$\le$.001
$\hbox {NO}_2$ max	Eyes	−0.573	$\le$.001
$\hbox {NO}_x$ mean	Nose	−0.5728	$\le$.001
$\hbox {NO}_2$ mean	Breathing	−0.5658	$\le$.001
$\hbox {SO}_2$ mean	Breathing	−0.5627	$\le$.001
$\hbox {NO}_x$ mean	Max_score	−0.56	$\le$.001
$\hbox {NO}_x$ max	Nose	-0.5468	$\le$.001
$\hbox {NO}_x$ max	Max_score	-0.5428	0.0011
$\hbox {NO}_x$ max	Taken_medication	-0.5326	0.0014
$\hbox {NO}_2$ max	Breathing	−0.5116	0.0023
$\hbox {NO}_x$ max	Breathing	−0.5024	0.0029
$\hbox {NO}_2$ mean	Max_score	−0.4904	0.0038
$\hbox {NO}_2$ mean	Nose	−0.4902	0.0038
$\hbox {NO}_x$ mean	Taken_medication	−0.4882	0.0039
$\hbox {SO}_2$ max	Eyes	−0.484	0.0043
$\hbox {NO}_2$ max	Max_score	−0.4693	0.0059
$\hbox {NO}_2$ max	Nose	−0.4652	0.0064
$\hbox {SO}_2$ mean	Max_score	−0.4538	0.008
Grass (Poaceae)	Max_score	0.4379	0.0108
$\hbox {O}_3$ max	Taken_medication	0.4268	0.0133
$\hbox {O}_3$ max	Nose	0.395	0.0229
Rel. hum. mean	Taken_medication	−0.384	0.0274
$\hbox {SO}_2$ max	Breathing	−0.3816	0.0285
Rel. hum. mean	Nose	−0.3792	0.0295
Grass (Poaceae)	Eyes	0.3751	0.0315
$\hbox {O}_3$ mean	Taken_medication	0.3729	0.0326
Temperature max	Breathing	0.3629	0.0379
$\hbox {O}_3$ mean	Nose	0.362	0.0385
$\hbox {NO}_2$ mean	Taken_medication	−0.3571	0.0413
Hazel (*Corylus* spp.)	Breathing	−0.3562	0.0419
$\hbox {NO}_2$ max	Taken_medication	−0.3558	0.0422
Rel. hum. mean	Max_score	−0.3548	0.0427

Environment measure (UK)	BB symptom (rural)	Correlation	p (2-tailed)
Grass (Poaceae)	Eyes	0.3852	0.0269
Grass (Poaceae)	Nose	0.3834	0.0276
$\hbox {SO}_2$ mean	Taken_medication	0.3663	0.036
Grass (Poaceae)	Max_score	0.3497	0.046

Only significant correlations (indicated by p values $\le$ 0.05) are included here.

Table 3 shows all statistically significant (p$\le$0.05) correlations between monthly means of BB symptoms and monthly means of environment measurements. The results are split between BB urban scores (top section) and BB rural scores (bottom section), and each section is ordered by significance. These results indicate that urban symptoms correlate more highly, and with a wider variety of UK-wide pollutant markers, than rural symptoms. The highest (absolute) monthly correlation is urban: a negative correlation of −0.72 between $\hbox {SO}_2$ (daily mean) and urban *eyes* symptoms. The strongest correlations for urban locations are for $\hbox {SO}_2$, $\hbox {NO}_x$, and $\hbox {NO}_2$. No symptoms exhibit a strong correlation with particulate matter pollutants ($\hbox {PM}_{10}$ and $\hbox {PM}_{2.5}$). The rural symptoms have only four significant correlations, with the highest correlation being +0.38 for *eyes* vs grass (Poaceae) (daily mean). The only factors rural symptoms correlate with are $\hbox {SO}_2$ and grass and, unlike in urban locations, no rural symptoms correlate with $\hbox {NO}_x$, $\hbox {NO}_2$, or $\hbox {O}_3$ factors. Although the highest rural symptom correlations are weaker than the highest urban symptom correlations, those factors common across both land-use types ($\hbox {SO}_2$ and grass) have similar magnitude correlations with symptoms.

Grass (Poaceae) pollen has similar positive correlations for both urban and rural areas and the only other pollen with significant correlation is hazel (*Corylus* spp.), but it is negative, perhaps due to its spring (February-March) peak, which would be inversely related to the later summertime peak of grass. Another pattern worthy of note is that all of the urban symptom correlations with pollutants are negative, except for $\hbox {O}_3$. Rural symptoms, on the other hand, only have positive correlations with one gaseous pollutant $\hbox {SO}_2$ (no significant correlations exist between rural symptoms and $\hbox {O}_3$).

Table 4 (top section) shows the correlations between average monthly urban BB symptom scores and *background urban* pollutant sensors. When BB urban scores are compared only with urban background sensors in this way, the scores remain very similar to those found when both urban and rural background sensors are used (Table 3). Once more, for urban locations, all correlations with $\hbox {O}_3$ are positive, and all correlations with other pollutants are negative. Again, the significant correlations are only with the gaseous pollutants; particulate matter ($\hbox {PM}_{10}$ and $\hbox {PM}_{2.5}$) show no significant correlations.

**Table 4:** Correlations of BB symptom scores with average monthly pollutant variables (UK-wide, but grouped by sensor location type, March–Sept incl.).

Environment measure (background urban)	BB symptom (urban)	Correlation	p (2-tailed)
$\hbox {SO}_2$ mean	Eyes	−0.713	$\le$.001
$\hbox {NO}_x$ mean	Eyes	−0.6596	$\le$.001
$\hbox {SO}_2$ mean	Nose	−0.633	$\le$.001
$\hbox {NO}_2$ mean	Eyes	$-$0.6237	$\le$.001
$\hbox {NO}_x$ max	Eyes	−0.6142	$\le$.001
$\hbox {NO}_x$ mean	Breathing	−0.604	$\le$.001
$\hbox {NO}_x$ mean	Nose	−0.5961	$\le$.001
$\hbox {NO}_2$ max	Eyes	−0.5948	$\le$.001
$\hbox {NO}_2$ mean	Breathing	−0.5819	$\le$.001
$\hbox {NO}_x$ mean	Max_score	−0.5687	$\le$.001
$\hbox {NO}_x$ max	Nose	−0.5662	$\le$.001
$\hbox {SO}_2$ mean	Breathing	−0.5524	$\le$.001
$\hbox {NO}_x$ max	Max_score	−0.5496	$\le$.001
$\hbox {NO}_x$ max	Taken_medication	−0.5384	0.0012
$\hbox {NO}_2$ max	Breathing	−0.5226	0.0018
$\hbox {NO}_2$ mean	Nose	−0.5205	0.0019
$\hbox {SO}_2$ max	Eyes	−0.5198	0.0019
$\hbox {NO}_x$ max	Breathing	−0.5083	0.0025
$\hbox {NO}_2$ mean	Max_score	−0.506	0.0027
$\hbox {NO}_x$ mean	Taken_medication	−0.4987	0.0031
$\hbox {NO}_2$ max	Nose	−0.4921	0.0036
$\hbox {NO}_2$ max	Max_score	−0.4812	0.0046
$\hbox {SO}_2$ mean	Max_score	−0.457	0.0075
$\hbox {O}_3$ max	Taken_medication	0.4355	0.0113
$\hbox {SO}_2$ max	Breathing	−0.4263	0.0134
$\hbox {O}_3$ max	Nose	0.4248	0.0137
$\hbox {O}_3$ mean	Nose	0.4131	0.0169
$\hbox {O}_3$ mean	Taken_medication	0.4031	0.02
$\hbox {SO}_2$ max	Nose	−0.3948	0.023
$\hbox {NO}_2$ mean	Taken_medication	−0.3752	0.0314
$\hbox {NO}_2$ max	Taken_medication	−0.3665	0.0359

Environment measure (background rural)	BB symptom (rural)		
$\hbox {SO}_2$ max	Eyes	0.4975	0.0032
$\hbox {SO}_2$ mean	Taken_medication	0.4935	0.0035
$\hbox {SO}_2$ mean	Max_score	0.4306	0.0124
$\hbox {SO}_2$ mean	Eyes	0.4136	0.0167
$\hbox {SO}_2$ max	Max_score	0.3861	0.0265
$\hbox {SO}_2$ mean	Nose	0.3603	0.0394
$\hbox {SO}_2$ max	Taken_medication	0.3591	0.0401
$\hbox {SO}_2$ max	Nose	0.358	0.0408

Only significant correlations (indicated by p values $\le$ 0.05) are included here.

Table 4 (bottom section) shows the correlations between average monthly rural BB symptom scores and *background rural* pollutant sensors. When BB rural scores are only compared with rural background sensors in this way, the highest rural correlation ($\hbox {SO}_2$ max daily and eyes) has risen to +0.5. Once more, all correlations with the $\hbox {SO}_2$ pollutant levels are positive, and no significant correlations exist with any other pollutants.

Overall, these correlations at the coarse-grained level, using monthly means and UK-wide environment variables, show that relationships exist between the pollution levels and allergy symptoms, but they are complex and will be explored further in the Discussion section.

### Regional-level correlations between BB symptoms and environment variables

To test for relationships between hay fever symptoms and environmental factors within more localised areas, we compare correlations between symptoms and pollen, pollutant and meteorological data at the regional level. Symptom reports (as in all previous analyses, these are limited to a maximum of 1 report per day per user, each including nose, eyes, breathing, and a *max score*: the maximum of these) are matched with mean environment measurements for the same postcode region. A concentric regions method^35^ (see “Methods” section, “Pre-processing” sub-section) is used to find environmental measurements where no sensors exist in a postcode area. This can lead to the use (in particular for pollen) of environmental data that is quite distant from the BB reports, and so we also calculate correlations for BB symptom reports only where environmental sensors for the given variable are available within the region. The correlations from both methods are presented for individual days, as well as aggregated for *weekly* and *monthly* periods (to allow for any potential lags between potential environmental triggers and symptoms). For this regional analysis, all pollution sensor types (urban background, rural background, industrial and urban traffic) are used.

**Table 5:** Correlations of BB symptom scores with environment variables, grouped by postcode region.

Time aggregation	Regions	Env variable	BB symptom	Correlation	p (2-tailed)
Daily mean	Concentric regions	Grass (Poaceae)	Max_score	**0.1139**	$\le$.001
		Grass	Eyes	0.109	$\le$.001
		Grass	Nose	0.1054	$\le$.001
	Same region only	Grass	Eyes	**0.1095**	$\le$.001
Weekly mean	Concentric regions	Grass	Max_score	**0.1653**	$\le$.001
		Grass	Nose	0.157	$\le$.001
		Grass	Eyes	0.1529	$\le$.001
		$\hbox {O}_3$ mean	Eyes	0.1113	$\le$.001
	Same region only	Grass	Eyes	**0.158**	$\le$.001
		$\hbox {SO}_2$ mean	Breathing	0.1541	$\le$.001
		Nettle family (Urticaceae)	Breathing	−0.1378	0.0023
		$\hbox {SO}_2$ mean	Taken_medication	0.1234	$\le$.001
		Grass	Nose	0.1212	0.0043
		$\hbox {SO}_2$ max	Taken_medication	0.1171	$\le$.001
		Grass	Max_score	0.1148	0.0068
Monthly mean	Concentric regions	Grass	Max_score	**0.1811**	$\le$.001
		Grass	Nose	0.1708	$\le$.001
		Grass	Eyes	0.165	$\le$.001
		$\hbox {O}_3$ mean	Eyes	0.1309	$\le$.001
		$\hbox {O}_3$ max	Taken_medication	0.1096	$\le$.001
		$\hbox {O}_3$ max	Eyes	0.1069	$\le$.001
	Same region only	$\hbox {SO}_2$ Mean	Breathing	**0.21**	$\le$.001
		Hazel (*Corylus* spp.)	Breathing	0.1888	0.0145
		Nettle family	Breathing	−0.1864	0.0156
		$\hbox {SO}_2$ mean	Max_score	0.1707	0.0015
		$\hbox {SO}_2$ max	Taken_medication	0.1665	0.002
		Grass	Eyes	0.1561	0.0311
		$\hbox {SO}_2$ mean	Taken_medication	0.1502	0.0054
		$\hbox {SO}_2$ mean	Nose	0.1297	0.0164
		$\hbox {SO}_2$ max	Nose	0.1268	0.019
		$\hbox {SO}_2$ max	Breathing	0.1158	0.0323
		$\hbox {SO}_2$ max	Max_score	0.1117	0.0389

Only significant correlations (indicated by p values $\le$ 0.05) are included here. Data is for March–Sept (inclusive). Highest results for each frequency/region type combination are highlighted in bold.

Table 5 shows correlations between environment scores (pollen, pollutants and weather) that have a significance of p (2-tailed) $\le$ 0.05. Note that none of the meteorological variables correlated with the required significance to be included in this table. The top 3 rows show correlations of *daily* BB scores with *daily mean* environment scores using concentric regions and, in this case, only one environmental measurement correlates significantly with any BB symptom. Grass (Poaceae) correlates with BB nose symptoms 0.1054, eye symptoms: 0.109 and max score: 0.1139 (all with p (2-tailed) $\le$ 0.001). Using correlations between BB symptom reports and environmental variables in the same region only, only one significant correlation, between *eyes* and grass, occurs (shown in row 4), with no increase in correlation. Repeating the same analysis for *weekly* and *monthly* time aggregations leads to improved correlations (shown in rows 5–8 and 16–21), while using environmental sensors in the same region only increased correlations at the monthly level (shown in rows 22–32) (but not at the weekly level; shown in rows 9–15). These aggregations also broaden the range of environmental variables with which symptoms are correlated. The highest correlation (using monthly means and only matching BB and environment data from the same region) was 0.21, for breathing with $\hbox {SO}_2$ mean. It should be noted that this correlation is lower than any significant correlation reported using the urban/rural grouping for symptom reports at UK-wide level, as described in previous sections. The lack of significant increase in correlations when replacing the concentric regions method with one that uses the *same region only*, to minimise this distance, suggests a number of possibilities: (a) the postcode regions used are too large, (b) the smaller sample size of BB reports with a sensor in the same region allows the results to be more influenced by noise, or (c) that other factors are at play. Previous research suggests there are a number of reasons which could make strong correlations unlikely. For example, they could be affected by the complex dynamics of atmospheric components^23,43,44^ or the spatial heterogeneity of environment factors^45,46^, across the postcode regions (and so between the nearest sensor and each BB report).

To illustrate the regional variability of atmospheric components, Table 6 shows how measurement sites for each pollutant, weather and pollen variable correlate within single regions (see bold for median regional correlations), and across the entire UK (see $\dagger$ for the median inter-sensor correlations for all sensor pairs across the UK). The first 9 measurements in the table all have 2 or more working sensors in at least one postcode region and at least one pair of those sensors has a correlation with a p value of p$\le$0.05. The strong correlations for these variables (and, in particular, for pressure, temperature, $\hbox {O}_3$, $\hbox {PM}_{10}$, and $\hbox {PM}_{2.5}$) between the sensor-pairs both regionally and UK-wide give us confidence that it is reasonable for us to use these sensor data to infer temporal patterns (at least) for these at the regional scale. The lowest performing measurement of this group is $\hbox {SO}_2$, which has only a median UK-wide correlation of 0.146. Only one region (Belfast) with more than one $\hbox {SO}_2$ sensor (with a correlation p value of p$\le$0.05) has three sensors and a median correlation of 0.219. An expanded version of Table 6, showing the regions with the lowest and highest median inter-sensor correlations , as well as the mean, minimum and maximum sensor-pair correlations for these regions, is presented in Supplementary Table S6. The last 12 rows (all pollen type measurements) do not show any regions containing more than one sensor, so only their UK correlations are displayed. In this latter group, the UK-wide correlations are also considerably lower than the first group, bringing their ability to represent the majority of BB user environment conditions into question: the highest pollen median correlation is grass (Poaceae), at 0.302.

**Table 6:** Pollution, pollen and weather measurement: median correlations both within single postcode regions (bold), and across the whole UK ($\dagger$).

Measurement	Region count (regions with $\ge$2 sensors)		Median corr
NO2_mean	44	Median	**0.689**
		UK (all regions)	0.531$\dagger$
NOXasNO2_mean	44	Median	**0.63**
		UK (all regions)	0.482$\dagger$
O3_mean	12	Median	**0.892**
		UK (all regions)	0.665$\dagger$
PM10_mean	28	Median	**0.853**
		UK (all regions)	0.678$\dagger$
PM2.5_mean	20	Median	**0.948**
		UK (all regions)	0.73$\dagger$
Pressure_mean	28	Median	**0.998**
		UK (all regions)	0.939$\dagger$
Relativehumidity_mean	67	Median	**0.85**
		UK (all regions)	0.461$\dagger$
SO2_mean	5	Median	**0.219**
		UK (all regions)	0.146$\dagger$
Temperature_mean	67	Median	**0.985**
		UK (all regions)	0.922$\dagger$
Alder (*Alnus* spp.)	0	UK (all regions)	0.281$\dagger$
Ragweed (*Ambrosia* spp.)	0	UK (all regions)	0.099$\dagger$
Mugwort (*Artemisia* spp.)	0	UK (all regions)	0.153$\dagger$
Birch (*Betula* spp.)	0	UK (all regions)	0.219$\dagger$
Hazel (*Corylus* spp.)	0	UK (all regions)	0.093$\dagger$
Ash (*Fraxinus* spp.)	0	UK (all regions)	0.167$\dagger$
Plane (*Platanus* spp.)	0	UK (all regions)	0.199$\dagger$
Grass (Poaceae)	0	UK (all regions)	0.302$\dagger$
Oak (*Quercus* spp.)	0	UK (all regions)	0.162$\dagger$
Willow (*Salix* spp.)	0	UK (all regions)	0.166$\dagger$
Elm (*Ulmus* spp.)	0	UK (all regions)	0.134$\dagger$
Nettle family (Urticaceae)	0	UK (all regions)	0.212$\dagger$

Data for March–September, inclusive, is used. Only significant correlations (those with p$\le$0.05) are reported here.

## Discussion

**Figure 3:**
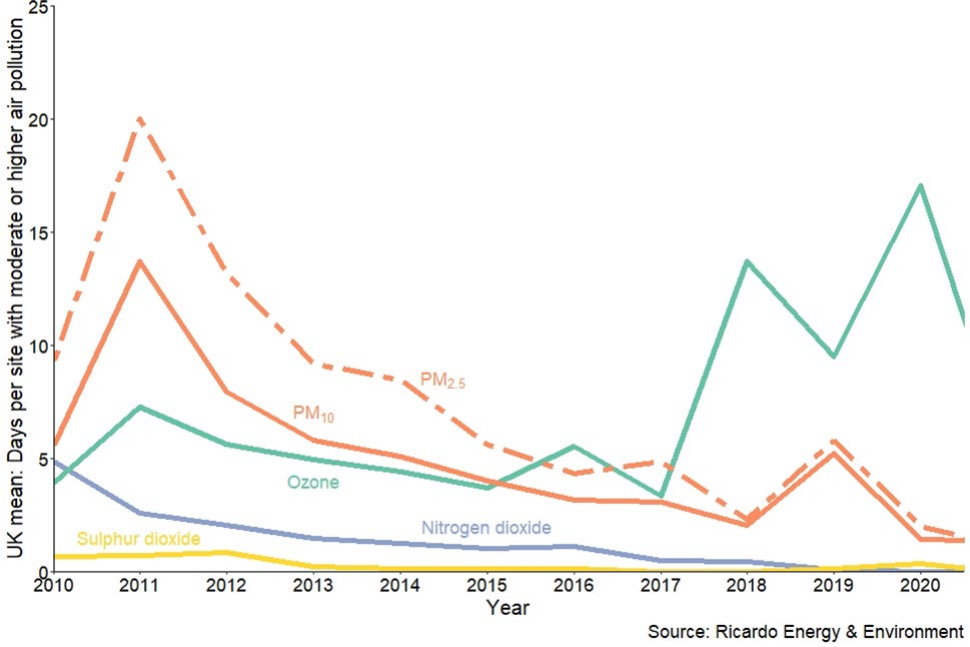
UK mean days per (urban) site with moderate or higher air pollution by year (from DEFRA report^47^).

**Figure 4:**
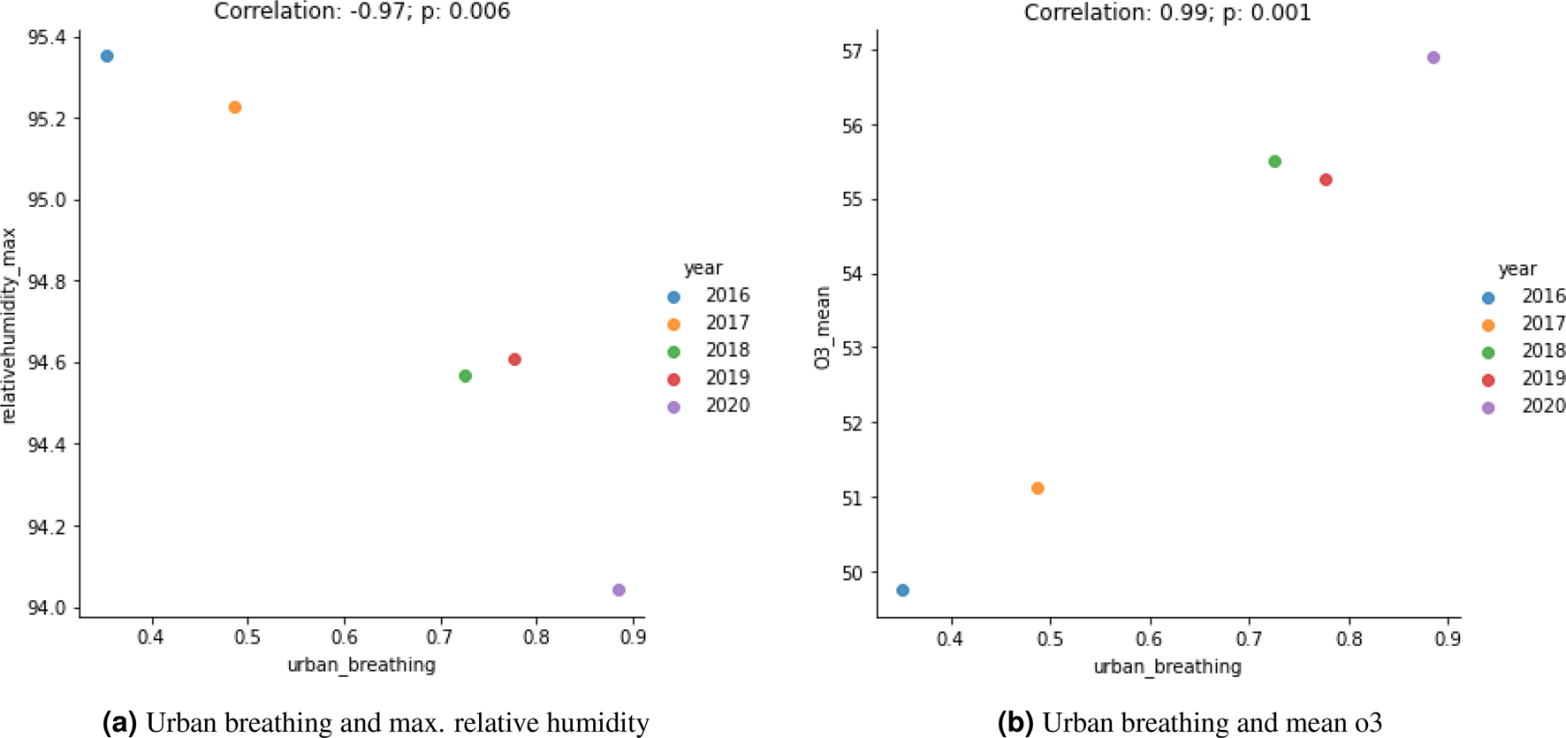
BB symptom *urban breathing* correlations with relative humidity (**a**) and mean $\hbox {O}_3$ (**b**), aggregated by year.

One potential reason for the reduced differences between urban and rural symptom severity in 2017, might be that according to DEFRA reports (see Fig. 3 taken from^47^, as well as^12^), the number of days with moderate or higher $\hbox {O}_3$ levels dropped slightly in 2017 before rising sharply and staying relatively high in subsequent years. Another factor worth considering is that 2017 was warmer and wetter than other years^48^. Temperature, precipitation and humidity have been found to have an effect on pollen counts^40,49–51^ and also potentially on pollution levels or participants’ biological reactions to such factors. Figure 4 displays the correlations between yearly means of urban BB symptoms with (a) relative humidity (daily max) and (b) $\hbox {O}_3$ (daily mean) respectively. The relative humidity correlation suggests that wetter weather could reduce the severity of some symptoms, or possibly that $\hbox {O}_3$ increases symptoms. Previous work also suggests that $\hbox {O}_3$ is associated with warmer weather^15^. The presence of any of the above types of phenomena could be (directly or indirectly) related to reductions in urban symptoms and/or the increase in rural symptoms, potentially lessening the gap between the two for that year.

It is worthy of note that there is a negative correlation between symptom severity and all gaseous pollutants in urban areas, except for $\hbox {O}_3$ which showed significant positive correlations. This indicates an inverse relationship, in urban areas, between ozone and other pollutants, which has been discussed in several recent studies highlighting the *weekend effect*^15,52,53^ where $\hbox {O}_3$ levels increase (often at weekends) as other pollutants reduce. The inverse relationship can also be seen in annual DEFRA figures (Fig. 3)^47^ in urban areas in years 2018 to 2020. We hypothesise that if any causal effect exists, is likely to be $\hbox {O}_3$ worsening symptoms, rather than other gaseous pollutants lessening them.

Weaker correlations between BB hay fever symptoms and environmental variables are observed at a regional level, using the postcode regions method (Table 5), which we suggest could be due to any combination of the following complex factors: the spatial mix of pollutant emission sources; interactions between atmospheric components^3^; and the spatial variability of pollens and some pollutants such as $\hbox {NO}_2$ and $\hbox {SO}_2$ (e.g.^45,46,54^), which will create distribution patterns that do not map cleanly onto postcode regions. Any relationships are likely to be further obfuscated by the variety of possible human immune responses.

In future Britain Breathing studies, we intend to obtain more information from users about whether they report symptoms before or after taking any antihistamines (using responses to the ’Taken Medication?’ question), so that we can stratify all symptom results by this factor. This would rule out any effects due to users’ interpretations of whether the symptoms should be recorded whilst such medications are active.

## Conclusions

The main aim of this study was to investigate the relationship between environmental factors and real-time hay fever symptom reports using experience sampled, cross sectional data from the general population^9^. To capture any potential relationship, we used a simple method of dividing and comparing symptoms according to whether they occur in urban or rural areas as these are (as outlined in the Introduction) reported to vary in pollen counts and types, pollution levels and rates of allergic reactions.

Our overall results indicate that H1 (*Seasonal allergy symptoms are worse for those in urban areas than in rural areas.*) is supported. When observing differences between rural and urban symptom severity, the associated Kolmogorov-Smirnov tests displayed in Table 1 show that in all years except 2017, urban means are significantly higher than rural means for *nose*, *taken_medication* and *max_score*. The bootstrap re-sampling method results, illustrated in Fig. 2 also show considerable differences across similar year symptom combinations, allowing confidence that these differences are not biased by a few individuals reporting from one land use type. This motivates further investigation into the reason why urban and rural symptom levels were more similar in 2017 and we suggest possible reasons for this in the Discussion section.

Symptom duration, measured in unbroken sets of days of reported symptoms, is also higher in urban locations, suggesting that symptoms in urban areas are not only likely to be more severe, but to also last longer.

The results of analyses performed to test H2 (*Higher levels of pollution lead to worse seasonal allergy symptoms.*) were less conclusive and indicate a complex relationship between environment variables and allergy symptoms. We have measured UK-wide correlations between hay fever symptoms and environmental factors and compared differences between urban and rural locations (Tables 3 and 4). Results indicate higher (moderate) correlations in urban areas. Whilst urban symptoms correlate more highly with all gaseous pollutants, rural symptoms correlate only with $\hbox {SO}_2$ and grass pollen.

## Supplementary Information

Supplementary Information 1.

Supplementary Information 2.

## Data Availability

All environmental data described in this publication are publicly available^33,34^. Britain Breathing data is not publicly accessible, due to the possibility of identifying individual participants from the location data. Should people have queries about this data or wish to obtain it under a data sharing agreement, they are invited to contact the lead author.
