# Supplementary material for: A comparison of experience sampled hay fever symptom severity across rural and urban areas of the UK
Source: Sci Rep. 2023 Feb 21;13:3060. doi: 10.1038/s41598-023-30027-x (PMC9944909; doi:10.1038/s41598-023-30027-x)
Supplement: Supplementary file 1 — Supplementary Information 1. [file 41598_2023_30027_MOESM1_ESM.pdf]

Appendix: A comparison of experience sampled hay fever symptom severity across rural and urban areas of the UK

Ann Gledson[1], Douglas Lowe[1], Manuele Reani[2], David Topping[3], Ian Hall[5], Sheena Cruickshank[6], Adrian Harwood[1], Joshua Woodcock[1], and Caroline Jay[4]

[1]Research IT, University of Manchester, UK
[2]School of Management and Economics, The Chinese University of Hong Kong, Shenzhen, China
[3]Department of Earth and Environmental Sciences, University of Manchester, UK
[4]Department of Computer Science, University of Manchester, UK
[5]Department of Mathematics, University of Manchester, UK
[6]Division of Infection, Immunity & Respiratory Medicine, University of Manchester, UK
[*]ann.gledson@manchester.ac.uk

Appendix

Land-use classification into urban and rural

Land-use data was obtained from the UK's Office for National Statistics (ONS)[1] and the ONS *2011 Census Rural-Urban Classification*[1]. The categories are as follows:

- England and Wales
  - A1 = urban major conurbation: OA falls within a built-up area with a population of 10,000 or more and is assigned to the 'major conurbation' settlement category. The wider surrounding area is less sparsely populated;
  - B1 = urban minor conurbation: OA falls within a built-up area with a population of 10,000 or more and is assigned to the 'minor conurbation' settlement category. The wider surrounding area is less sparsely populated;
  - C1 = urban city and town: OA falls within a built-up area with a population of 10,000 or more and is assigned to the 'city and town' settlement category. The wider surrounding area is less sparsely populated;
  - C2 = urban city and town in a sparse setting: OA falls within a built-up area with a population of 10,000 or more and is assigned to the 'city and town' settlement category. The wider surrounding area is sparsely populated;
  - D1 = rural town and fringe: OA is assigned to the 'town and fringe' settlement category. The wider surrounding area is less sparsely populated;
  - D2 = rural town and fringe in a sparse setting: OA is assigned to the 'town and fringe' settlement category. The wider surrounding area is sparsely populated;
  - E1 = rural village: OA is assigned to the 'village' settlement category. The wider surrounding area is less sparsely populated;
  - E2 = rural village in a sparse setting: OA is assigned to the 'village' settlement category. The wider surrounding area is sparsely populated;
  - F1 = rural hamlet and isolated dwellings: OA is assigned to the 'hamlet and isolated dwelling' settlement category. The wider surrounding area is less sparsely populated;
  - F2 = rural hamlet and isolated dwellings in a sparse setting: OA is assigned to the 'hamlet and isolated dwelling' settlement category. The wider surrounding area is sparsely populated.
- Scotland
  - 1 = Large Urban Area: Settlement of over 125,000 people;

- 2 = Other Urban Area: Settlement of 10,000 to 125,000 people;
- 3 = Accessible Small Town: Settlement of 3,000 to 10,000 people, within 30 minutes' drive of a settlement of 10,000 or more;
- 4 = Remote Small Town: Settlement of 3,000 to 10,000 people, with a drive time of 30 to 60 minutes to a settlement of 10,000 or more;
- 5 = Very Remote Small Town: Settlement of 3,000 to 10,000 people, with a drive time of over 60 minutes to a settlement of 10,000 or more;
- 6 = Accessible Rural: Settlement of less than 3,000 people, within 30 minutes' drive of a settlement of 10,000 or more;
- 7 = Remote Rural: Settlement of less than 3,000 people, with a drive time of 30 to 60 minutes to a settlement of 10,000 or more;
- 8 = Very Remote Rural: Settlement of less than 3,000 people, with a drive time of over 60 minutes to a settlement of 10,000 or more.

References

1. Office for National Statistics. *National Statistics Postcode Lookup User Guide*.
