# Supplementary material for: A comparison of experience sampled hay fever symptom severity across rural and urban areas of the UK
Source: Sci Rep. 2023 Feb 21;13:3060. doi: 10.1038/s41598-023-30027-x (PMC9944909; doi:10.1038/s41598-023-30027-x)
Supplement: Supplementary file 2 — Supplementary Information 2. [file 41598_2023_30027_MOESM2_ESM.pdf]

SUPPLEMENTARY MATERIAL: A comparison of experience sampled hay fever symptom severity across rural and urban areas of the UK

Ann Gledson[1], Douglas Lowe[1], Manuele Reani[2], David Topping[3], Ian Hall[5], Sheena Cruickshank[6], Adrian Harwood[1], Joshua Woodcock[1], and Caroline Jay[4]

[1]Research IT, University of Manchester, UK
[2]School of Management and Economics, The Chinese University of Hong Kong, Shenzhen, China
[3]Department of Earth and Environmental Sciences, University of Manchester, UK
[4]Department of Computer Science, University of Manchester, UK
[5]Department of Mathematics, University of Manchester, UK
[6]Division of Infection, Immunity & Respiratory Medicine, University of Manchester, UK
[*]ann.gledson@manchester.ac.uk

We compared mean daily symptom severity between land-use types. Reports were classified as urban or rural and we calculated the mean scores for each day, for each location type and symptom combination. These mean scores are then aggregated by year (2016 to 2020). Table S1a shows comparisons between urban and rural mean scores for each symptom (or whether medication was taken), for all months of the year (See Supplementary Table S1b for comparisons using only BB reports from March-September incl., in which the differences are as pronounced, if not more so.). The first row displays the averages across all years, and the remaining rows display each year. Nested rows show data for each symptom. The *diff mean* column is the *urban mean* score minus the *rural mean* score, so positive values indicate a higher urban mean score. Urban and rural standard deviations and the difference in standard deviations, are also shown. We used *Cohen's d*? to measure the effect size between the two means. Effect sizes can be categorised as: 0.01=very small; 0.2=small; 0.5=medium; 0.8=large; 1.2=very large; 2.0=huge. Table S1a also displays the non-parametric *Kolmogorov-Smirnov* test result (U1), used to compare the distance between the urban and rural daily mean distributions. Higher scores represent a greater difference between the two distribution functions, with a possible range of 0 to 1.

	BB measure	urban mean	rural mean	urban std	rural std	% diff std	diff mean	cohen's d	U1	p (2-tailed)
All years	nose	0.738	0.376	0.412	0.404	0.02	0.362	0.887	0.406	<.001***
	eyes	0.614	0.372	0.416	0.43	0.03	0.241	0.57	0.352	<.001***
	breathing	0.63	0.331	0.483	0.357	0.26	0.299	0.7	0.293	<.001***
	taken_meds	0.63	0.397	0.248	0.32	0.23	0.233	0.818	0.310	<.001***
	max_score	1.037	0.614	0.468	0.534	0.12	0.423	0.843	0.35	<.001***
2016	nose	0.566	0.315	0.166	0.245	0.32	0.251	1.2	0.538	<.001***
	eyes	0.429	0.296	0.149	0.227	0.34	0.133	0.692	0.396	<.001***
	breathing	0.336	0.389	0.098	0.196	0.5	-0.052	-0.338	0.271	<.001***
	taken_meds	0.496	0.51	0.088	0.171	0.49	-0.014	-0.102	0.196	<.001***
	max_score	0.786	0.649	0.173	0.275	0.37	0.136	0.592	0.342	<.001***
2017	nose	0.626	0.6	0.251	0.335	0.25	0.027	0.09	0.136	.0223*
	eyes	0.484	0.541	0.242	0.322	0.25	-0.057	-0.202	0.185	<.001***
	breathing	0.398	0.459	0.222	0.259	0.14	-0.061	-0.253	0.174	.0013**
	taken_meds	0.574	0.607	0.176	0.218	0.19	-0.032	-0.163	0.149	.009**
	max_score	0.911	0.918	0.298	0.365	0.18	-0.007	-0.021	0.142	.0147*
2018	nose	0.705	0.592	0.29	0.35	0.17	0.113	0.352	0.169	<.001***
	eyes	0.598	0.604	0.31	0.43	0.28	-0.006	-0.015	0.093	.0795 ns
	breathing	0.57	0.545	0.327	0.327	0	0.025	0.077	0.112	.019*
	taken_meds	0.703	0.593	0.141	0.244	0.42	0.11	0.55	0.287	<.001***
	max_score	1.044	0.931	0.381	0.452	0.16	0.113	0.271	0.177	<.001***
2019	nose	0.593	0.28	0.439	0.439	0	0.314	0.713	0.423	<.001***
	eyes	0.5	0.349	0.419	0.517	0.19	0.151	0.321	0.349	<.001***
	breathing	0.668	0.195	0.585	0.37	0.37	0.473	0.959	0.429	<.001***
	taken_meds	0.654	0.282	0.332	0.318	0.04	0.372	1.142	0.435	<.001***
	max_score	0.972	0.443	0.597	0.581	0.03	0.529	0.897	0.428	<.001***
2020	nose	1.057	0.12	0.503	0.352	0.3	0.937	2.123	0.821	<.001***
	eyes	0.957	0.1	0.512	0.314	0.39	0.857	1.975	0.815	<.001***
	breathing	0.963	0.133	0.544	0.358	0.34	0.83	1.769	0.772	<.001***
	taken_meds	0.625	0.095	0.289	0.219	0.24	0.53	2.036	0.754	<.001***
	max_score	1.287	0.215	0.499	0.469	0.06	1.072	2.205	0.784	<.001***

Supplementary Table S1a. Urban vs Rural symptom severity (all months). Average daily scores for each are presented, with the difference between the two (diff), the Kolmogorov-Smirnov test statistic (U1), and significance (p). p values > 0.05 are marked as *ns*.

	BB measure	urban mean	rural mean	urban std	rural std	% diff std	diff mean	cohen's d	U1	p (2-tailed)
**All years**	nose	0.787	0.426	0.375	0.408	0.08	0.361	0.921	0.413	<.001***
	eyes	0.692	0.42	0.405	0.412	0.02	0.272	0.665	0.343	<.001***
	breathing	0.656	0.375	0.409	0.373	0.09	0.281	0.716	0.314	<.001***
	taken_meds	0.647	0.458	0.224	0.314	0.29	0.188	0.69	0.254	<.001***
	max_score	1.103	0.683	0.414	0.529	0.22	0.42	0.884	0.387	<.001***
**2016**	nose	0.603	0.342	0.142	0.249	0.43	0.261	1.284	0.571	<.001***
	eyes	0.466	0.33	0.123	0.223	0.45	0.136	0.752	0.429	<.001***
	breathing	0.346	0.403	0.088	0.198	0.56	-0.057	-0.375	0.304	<.001***
	taken_meds	0.51	0.538	0.081	0.159	0.49	-0.028	-0.218	0.22	<.001***
	max_score	0.817	0.688	0.156	0.27	0.42	0.129	0.584	0.361	<.001***
**2017**	nose	0.709	0.742	0.246	0.275	0.11	-0.033	-0.125	0.143	.0915 ns
	eyes	0.57	0.647	0.254	0.291	0.13	-0.077	-0.282	0.178	.018*
	breathing	0.467	0.505	0.216	0.256	0.16	-0.038	-0.163	0.217	0.0018**
	taken_meds	0.53	0.681	0.176	0.205	0.14	-0.15	-0.786	0.376	<.001***
	max_score	1.022	1.053	0.284	0.33	0.14	-0.031	-0.099	0.19	0.0095**
**2018**	nose	0.828	0.667	0.261	0.305	0.14	0.161	0.567	0.25	<.001***
	eyes	0.743	0.673	0.28	0.354	0.21	0.07	0.218	0.165	0.0053**
	breathing	0.699	0.623	0.259	0.304	0.15	0.076	0.268	0.176	0.0022**
	taken_meds	0.723	0.668	0.128	0.195	0.34	0.055	0.331	0.184	0.0013**
	max_score	1.23	1.022	0.291	0.374	0.22	0.208	0.619	0.297	<.001***
**2019**	nose	0.653	0.311	0.431	0.431	0	0.341	0.79	0.384	<.001***
	eyes	0.563	0.356	0.453	0.48	0.06	0.207	0.443	0.323	<.001***
	breathing	0.727	0.223	0.499	0.407	0.18	0.504	1.104	0.527	<.001***
	taken_meds	0.715	0.351	0.301	0.315	0.04	0.364	1.18	0.499	<.001***
	max_score	1.097	0.507	0.55	0.558	0.01	0.59	1.064	0.462	<.001***
**2020**	nose	1.097	0.145	0.43	0.399	0.07	0.952	2.285	0.853	<.001***
	eyes	1.055	0.142	0.455	0.369	0.19	0.912	2.188	0.811	<.001***
	breathing	0.954	0.147	0.457	0.397	0.13	0.807	1.876	0.792	<.001***
	taken_meds	0.707	0.111	0.241	0.233	0.03	0.596	2.508	0.774	<.001***
	max_score	1.293	0.237	0.443	0.519	0.15	1.057	2.191	0.791	<.001***

Supplementary Table S1b. Urban vs Rural symptom severity (March-Sept incl.). Average daily scores for each are presented, with the difference between the two (diff), the Kolmogorov-Smirnov test statistic (U1), and significance (p). p values > 0.05 are marked as *ns*.

measurement	region count (regions with $\geq$2 sensors)		region_id	BB reports	sensors	mean distance (m)	mean corr	min corr	max corr	median corr
NO_2 mean	44	lowest	AB	170	3	936	0.452	0.274	0.75	0.333
		median								**0.689**
		highest	B	570	5	4917	0.826	0.665	0.938	0.845
		UK (all regions)		27798	161	258031	0.523	-0.143	0.952	0.531 †
NO_x mean	44	lowest	AB	170	3	936	0.457	0.286	0.687	0.399
		median								**0.63**
		highest	B	570	5	4917	0.77	0.53	0.907	0.826
		UK (all regions)		27798	161	258031	0.477	-0.251	0.947	0.482 †
O_3 mean	12	lowest	BT	134	3	50426	0.847	0.786	0.921	0.835
		median								**0.892**
		highest	B	570	3	2674	0.922	0.917	0.931	0.919
		UK (all regions)		27798	75	283791	0.655	0.186	0.958	0.665 †
$PM1_{10}$ mean	28	lowest	SA	61	3	22464	0.614	0.444	0.797	0.602
		median								**0.853**
		highest	B	570	3	2674	0.975	0.968	0.985	0.972
		UK (all regions)		27798	90	256115	0.661	0.143	0.988	0.678 †
$PM_{2.5}$ mean	20	lowest	BT	134	3	50426	0.769	0.691	0.821	0.794
		median								**0.948**
		highest	B	570	3	2674	0.977	0.962	0.985	0.983
		UK (all regions)		27798	81	260858	0.71	0.19	0.985	0.73 †
pressure mean	28	lowest	PA	16	6	123793	0.979	0.945	0.999	0.986
		median								**0.998**
		highest	EX	772	4	27474	0.998	0.996	0.999	0.999
		UK (all regions)		27798	154	373223	0.915	0.424	1	0.939 †
rel. humid. mean	67	lowest	BS	472	3	7130	0.549	0.156	0.942	0.549
		median								**0.85**
		highest	TW	110	4	4975	0.978	0.964	0.989	0.98
		UK (all regions)		27798	323	355243	0.47	-0.292	0.998	0.461 †
SO_2 mean	5	lowest	BT	134	3	34613	0.219	0.076	0.362	0.219
		median								**0.219**
		highest	BT	134	3	34613	0.219	0.076	0.362	0.219
		UK (all regions)		27798	28	253280	0.142	-0.289	0.483	0.146 †
temperature mean	67	lowest	BS	472	3	7130	0.794	0.69	0.997	0.696
		median								**0.985**
		highest	TW	110	4	4975	0.997	0.995	0.999	0.998
		UK (all regions)		27798	323	355243	0.914	0.542	1	0.922 †
alder (*Alnus* spp.)	0	UK (all regions)		27798	13	273984	0.309	0.074	0.808	0.281 †
ragweed (*Ambrosia* spp.)	0	UK (all regions)		27798	13	273984	0.141	0.074	0.371	0.099 †
mugwort (*Artemisia* spp.)	0	UK (all regions)		27798	13	273984	0.176	0.079	0.423	0.153 †
birch (*Betula* spp.)	0	UK (all regions)		27798	13	273984	0.243	0.077	0.767	0.219 †
hazel (*Corylus* spp.)	0	UK (all regions)		27798	13	273984	0.161	0.08	0.617	0.093 †
ash (*Fraxinus* spp.)	0	UK (all regions)		27798	13	273984	0.212	0.069	0.651	0.167 †
plane (*Platanus* spp.)	0	UK (all regions)		27798	13	273984	0.239	0.083	0.496	0.199 †
grass (Poaceae)	0	UK (all regions)		27798	15	260237	0.284	-0.222	0.739	0.302 †
oak (*Quercus* spp.)	0	UK (all regions)		27798	13	273984	0.212	0.07	0.524	0.162 †
willow (*Salix* spp.)	0	UK (all regions)		27798	13	273984	0.188	0.072	0.488	0.166 †
elm (*Ulmus* spp.)	0	UK (all regions)		27798	13	273984	0.166	0.075	0.469	0.134 †
nettle family (Urticaceae)	0	UK (all regions)		27798	13	273984	0.259	-0.122	0.964	0.212 †

Supplementary Table S6. Pollution, pollen and weather measurement correlations both within single postcode regions (bold), and across the whole UK (†). Data for March-September, inclusive, is used. Only significant correlations (those with $p \leq 0.05$) are reported here.

To illustrate the regional variability of atmospheric components, Supplementary Table S6 shows how measurement sites for each pollutant, weather and pollen variable correlate within single regions (see bold for median regional correlations), and across the entire UK (see † for the median inter-sensor correlations for all sensor pairs across the UK). The first 9 measurements (outer rows) in the table all have 2 or more working sensors in at least one postcode region and at least one pair of those sensors has a correlation with a p value of $p \leq 0.05$. The regions with the lowest and highest median inter-sensor correlations are shown (nested rows), as well as the mean, minimum and maximum sensor-pair correlations for these regions (columns 8–10). In addition, the median of all regional inter-sensor correlation medians for that measurement are shown (final column). A further (nested) row is added for each measurement showing the median inter-sensor correlations for all sensor pairs across the UK (which also has columns for the minimum, maximum, and mean sensor-pair correlations). The strong correlations for these variables (and, in particular, for pressure, temperature, O_3, PM_{10}, and $PM_{2.5}$) between the sensor-pairs both regionally and UK-wide give us confidence that it is reasonable for us to use these sensor data to infer temporal patterns (at least) for these at the regional scale. The lowest performing measurement of this group is SO_2, which has only a median UK-wide correlation of

0.146. The only region with more than one SO_2 sensor (with a correlation p value of $p \leq 0.05$) is BT (Belfast), which has 3 sensors and a median correlation of 0.219. The last 12 rows (all pollen type measurements) do not show any regions containing more than one sensor, so only their UK correlations are displayed. In this latter group, the UK-wide correlations are also considerably lower than the first group, bringing their ability to represent the majority of BB user environment conditions into question: the highest pollen median correlation is grass (Poaceae), at 0.302.
